# Quantitative comparison of pre-treatment predictive and post-treatment measured dosimetry for selective internal radiation therapy using cone-beam CT for tumor and liver perfusion territory definition

**DOI:** 10.1186/s13550-020-00675-5

**Published:** 2020-08-14

**Authors:** Esmaeel Jafargholi Rangraz, Xikai Tang, Charlotte Van Laeken, Geert Maleux, Jeroen Dekervel, Eric Van Cutsem, Chris Verslype, Kristof Baete, Johan Nuyts, Christophe M. Deroose

**Affiliations:** 1 Nuclear Medicine, University Hospitals Leuven, Nuclear Medicine and Molecular Imaging, Department of Imaging & Pathology, Leuven, Belgium; 2 Radiology Section, University Hospitals Leuven, Department of Imaging and Pathology, Leuven, Belgium; 3 Digestive Oncology, University Hospitals Leuven, Leuven, Belgium

**Keywords:** Radioembolization, Selective internal radiation therapy (SIRT), Trans arterial radioembolization (TARE), Dose estimation, Dosimetry, Liver perfusion territory segmentation, CBCT, Dose validation, Dose comparison

## Abstract

**Background:**

Selective internal radiation therapy (SIRT) is a promising treatment for unresectable hepatic malignancies. Predictive dose calculation based on a simulation using ^99*m*^Tc-labeled macro-aggregated albumin (^99*m*^Tc-MAA) before the treatment is considered as a potential tool for patient-specific treatment planning. Post-treatment dose measurement is mainly performed to confirm the planned absorbed dose to the tumor and non-tumor liver volumes. This study compared the predicted and measured absorbed dose distributions.

**Methods:**

Thirty-one patients (67 tumors) treated by SIRT with resin microspheres were analyzed. Predicted and delivered absorbed dose was calculated using ^99*m*^Tc-MAA-SPECT and ^90^Y-TOF-PET imaging. The voxel-level dose distribution was derived using the local deposition model. Liver perfusion territories and tumors have been delineated on contrast-enhanced CBCT images, which have been acquired during the ^99*m*^Tc-MAA work-up. Several dose-volume histogram (DVH) parameters together with the mean dose for liver perfusion territories and non-tumoral and tumoral compartments were evaluated.

**Results:**

A strong correlation between the predicted and measured mean dose for non-tumoral volume was observed (*r* = 0.937). The ratio of measured and predicted mean dose to this volume has a first, second, and third interquartile range of 0.83, 1.05, and 1.25. The difference between the measured and predicted mean dose did not exceed 11 Gy. The correlation between predicted and measured mean dose to the tumor was moderate (*r* = 0.623) with a mean difference of − 9.3 Gy. The ratio of measured and predicted tumor mean dose had a median of 1.01 with the first and third interquartile ranges of 0.58 and 1.59, respectively. Our results suggest that ^99*m*^Tc-MAA-based dosimetry could predict under or over dosing of the non-tumoral liver parenchyma for almost all cases. For more than two thirds of the tumors, a predictive absorbed dose correctly indicated either good tumor dose coverage or under-dosing of the tumor.

**Conclusion:**

Our results highlight the predictive value of ^99*m*^Tc-MAA-based dose estimation to predict non-tumor liver irradiation, which can be applied to prescribe an optimized activity aiming at avoiding liver toxicity. Compared to non-tumoral tissue, a poorer agreement between predicted and measured absorbed dose is observed for tumors.

## Introduction

Selective internal radiation therapy (SIRT) is an increasingly applied palliative treatment option for unresectable primary and secondary hepatic malignancies. This treatment modality consists of infusing microspheres labeled with either yttrium-90 or holmium-166 within a selected branch of the hepatic artery. Choosing a proper branch of the hepatic artery is usually performed in the course of an angiography session, which aims at finding the tumor-feeding vessel(s) [1]. By infusing hundred-thousands/millions of microspheres through the tumor-feeding microvessels, they will get trapped within the liver, and the concentration of the lodged beads will be superior within the tumor compared to the non-tumoral liver parenchyma. The high energy and low tissue penetration of the used *β*-emitter (^90^Y or ^166^Ho) lead to higher energy deposited within the tumor compared to the non-tumoral hepatic tissue [2].

Recent studies showed a relation between tumor absorbed dose and tumor control probability, as well as non-tumoral liver absorbed dose and normal tissue complication probability. These insights serve as the basis of precision SIRT, where the prescribed injected activity is determined based on accurate knowledge of the biodistribution of the microspheres. The role of predictive dosimetry is becoming important in SIRT, and many recent studies have indicated to use a multi-compartment or voxel-level predictive dosimetry for this treatment. Ideally, dosimetric assessment should be performed in two steps: (1) absorbed dose prediction before treatment for each liver perfusion territory (LPT) which can be applied in an individual treatment planning to prescribe a tailored injected activity using patient-specific dosimetric criteria for liver perfusion territory (LPT), tumor volume (TV), and/or non-tumoral volume (NTV) and (2) absorbed dose evaluation after treatment for each LPT to determine the actual doses that have been given.

By definition, the absorbed dose is the amount of energy per mass (in Gy or $\frac {\mathrm {J}}{\text {kg}}$) delivered to a defined volume of interest (VOI), e.g., total liver, LPTs, NTVs, and TVs. To estimate the absorbed dose before treatment, a simulation is performed using ^99*m*^Tc-labeled macro-aggregated albumin (^99*m*^Tc-MAA) particles that mimic the intra- and extrahepatic biodistribution of the therapeutic microspheres, which are usually derived from a SPECT/CT image performed shortly after administration. Besides, after administrating the ^90^Y-microspheres, a Bremsstrahlung SPECT/CT and/or PET/CT or PET/MR image is performed which represents the actual activity distribution within the liver which again can be translated to an absorbed dose in each defined volume [3, 4].

Several studies showed a good correlation between pre-treatment dose estimation (using SPECT images from a ^99*m*^Tc-MAA study) and post-treatment dose calculation (using ^90^Y-PET images after treatment). On the other hand, some authors suggest that in certain circumstances, ^99*m*^Tc-MAA based dosimetry poorly predicts the actual absorbed dose. Cremonesi et al. provide some useful insight into the variations of existing SIRT dosimetry [5]. Several factors can describe this discrepancy: (a) the fundamental difference between ^99*m*^Tc-MAA and microspheres characteristics (e.g., size, morphology, density), (b) the difference between administration during the work-up and treatment session (e.g., catheter tip position, arterial vasospasm, and physiologic variances in hepatic blood flow), and (c) different VOI definition for predictive and post-therapy measured dosimetry.

One of the main contributors to over- or underestimation of the predicted and measured doses is the method used for VOI definition. In many studies, fixed or tumor-specific ^99*m*^Tc-MAA thresholding is used for TV definition [6, 7]. By thresholding the activity map, one considers that tumors correspond to high uptake regions, while the low-activity areas correspond to the non-tumoral liver compartment, which can be questionable if some fraction of the tumor has low uptake (which results in an overestimation of the tumor dose) or if some part of the non-tumoral tissue has a high activity accumulation (underestimation of non-tumoral liver dose) in pre- and/or post-treatment session. Also, this approach might reinforce the correlation between predicted and measured doses. Besides, the LPT definition plays an essential role in the VOI definition for extracting relevant dose reports, especially tumor to non-tumoral activity concentration ratio, tumor to non-tumoral volume ratio within the LPT, and accurate NTV definition for reporting doses. Typically, LPTs are segmented on anatomical images (CT or MR) using anatomical landmarks, which do not necessarily represent the volume that is irrigated by the branch of the hepatic artery tree that will be injected. Our previous study identified this discrepancy as a significant source of uncertainty while reporting the mean predicted dose for each LPT [8].

This study aims to evaluate the use of specific VOI definition (based on catheterization during pre-treatment angiographic work-up) for comparing predictive dosimetry and post-treatment dose measurement. In this study, contrast-enhanced cone-beam CT (CBCT) images, which are obtained with the catheter in different positions of the hepatic arterial tree, are used to define LPTs and tumors. After aligning these VOIs to the ^99*m*^Tc-MAA-SPECT and ^90^Y-PET space, the predicted and measured dose distribution within the tumor and non-tumoral liver parenchyma are reported. To our knowledge, this is the first study applying the CBCT-based VOI segmentation (most importantly for LPT segmentation) to validate ^99*m*^Tc-MAA-based dose estimation. Evaluating the correlation between predicted and measured dose is an important step towards precision SIRT and optimizing the likelihood of tumor response while minimizing the risk of normal liver complications.

## Materials and methods

### Patients

This retrospective study analyzed 31 patients out of a total of 49 treated patients with ^90^Y-labelled resin microspheres (SIR-Spheres, SIRTEX Medical Ltd, Sydney, Australia), between November 2017 and April 2019. This time frame was chosen because post-therapy imaging was performed with ^90^Y-PET since November 2017 in our center. Exclusion criteria were missing data (e.g., missing images), insufficient information (e.g., contrast enhancement in CBCT images), and patients without a tumor larger than 5 ml. This study was approved by the local University Hospital Ethics Committee (UZ/KU Leuven).

### Treatment workflow

All procedures were performed according to the *European Association of Nuclear Medicine (EANM)* guideline [9] and the recommendations of the *American Association of Physics in Medicine (AAPM)* [10].

In short, before treatment, all patients underwent a simulation work-up. During this session, an angiography was performed to identify the hepatic arterial anatomy, followed by a pair of contrast-enhanced CBCT focusing on each LPT in the early and late arterial phase. These interventional X-ray images were obtained using XtraVision (Philips Healthcare, Amsterdam, Netherlands). The CBCTs were performed by acquiring 60 frames per second while rotating the C-arm around the patient in around 5 to 8 s, using 120 kV tube voltage, 188 mA tube current, and tube current and voltage modulation. For early arterial phase scan, a delay of 6 s after initiation of the contrast medium injection was used. Then, the late arterial phase scan was performed with an 8-s delay after the end of the early arterial scan. The 2D projection images were reconstructed using standard vendor software with 0.66 mm isotropic resolution and matrix size of 384 ×384×297 pixels. Thereafter, ^99*m*^Tc-MAA particles were administered as slowly as possible while taking care to place the catheter at the exact same position as where it will be placed for treatment. As soon as possible after the administration, a planar gamma camera imaging and a SPECT/CT on a Symbia T16 dual-head gamma camera (Siemens Healthineers, Erlangen, Germany) were acquired to evaluate the possible lung shunt fraction (LSF) [11] and activity distribution within the liver. SPECT images were acquired with low-energy, high-resolution collimators with rotation over 180 ^∘^, 60 views per detector, and 21 s per view at an energy of 140 keV with a 15% energy window. These projections were reconstructed on a 128 ×128 matrix in an isotropic voxel size of 4.8 mm using ordered subset expectation maximization algorithm accounting for attenuation, position-dependent collimator blurring, a scatter contribution, which was estimated using a dual-energy scatter window, and a Gaussian post-reconstruction filter of 7.5 mm in full width at half maximum.

Before the treatment, baseline contrast-enhanced CT and/or MR imaging was performed for volumetric assessment (NTV and TV in each perfusion territory). By using this volumetric information together with the activity uptake information extracted from the ^99*m*^Tc-MAA study, an injected activity was prescribed for each LPT by applying either the MIRD model (non-compartmental partition model) or compartmental partition model using a conversion factor of 49.87 $\frac {\text {Gy}\times {\mathrm {{kg}}}}{\text {GBq}}$ aiming at patient-specific absorbed dose criteria for whole LPT (MIRD approach) or tumor compartment and non-tumoral liver parenchyma compartment. The dose to the lungs was kept below 30 Gy, using the calculated LSF on planar images at face value.

On the day of treatment, for each LPT, the catheter tip was in the same position as during the pre-treatment work-up. A day after administering the prescribed activity, the actual distribution of the microspheres was controlled by a time-of-flight (TOF)-PET examination on either a PET/MR system (Signa, GE Healthcare, Waukesha, MI, USA) or a PET/CT system (Discovery MI, GE Healthcare, Waukesha, MI, USA). The emission data were corrected for randoms, scatter, attenuation, TOF offset, and dead-time, and reconstructed using maximum-likelihood expectation-maximization algorithm using two iterations and 28 subsets. For PET imaging, the standard vendor attenuation correction was used: the attenuation map is estimated from the Dixon MR images (the Dixon sequence is called LAVA-FLEX) and CT image for the Signa and Discovery systems, respectively. For PET/CT system, a Gaussian post-reconstruction filter of 5 mm full-width at half-maximum in the *x*- and *y*-direction and a smoothing filter with coefficients [1,2,1] was applied in the *z*-direction. The reconstruction voxels have a dimension of 2.73 ×2.73×2.79 mm^3^. For PET/MR system, voxels with 3.13 ×3.13×2.78 mm^3^ dimensions were smoothed with a Gaussian filter with 7 mm full-width at half-maximum in the *x*- and *y*-direction and a [1,2,1] filter in the *z*-direction.

**Fig. 1:**
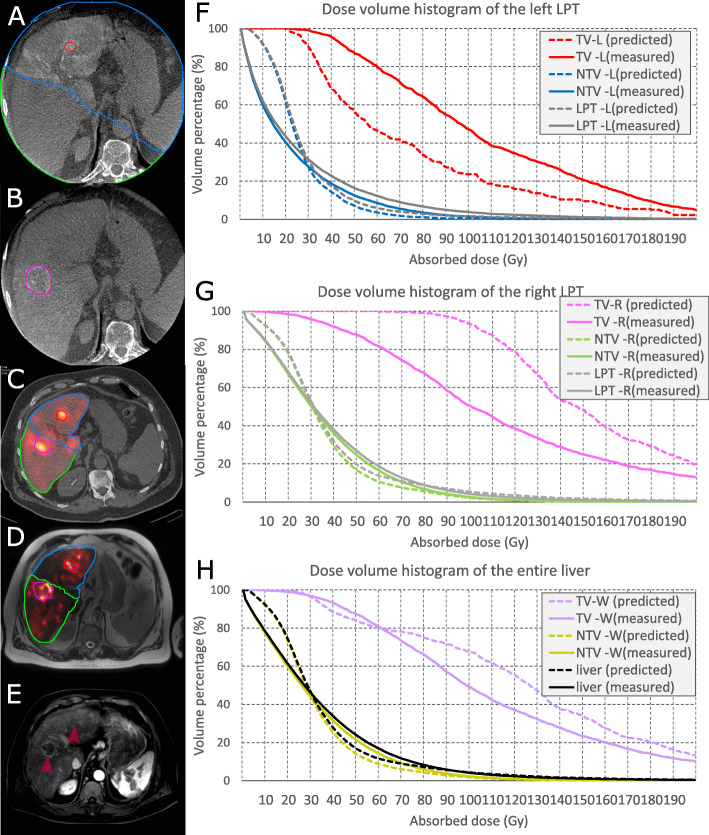
Single transaxial slice of a pre- and post-treatment study and dosimetry results (patient ID 42). **a**, **b** Late arterial phase CBCTs focusing on the left and right lobe; the contrast enhancement is used to segment the right and left LPT (green and blue area) and to segment the tumors (red and pink contours). **c**, **d** A fusion-view of ^99m^Tc-MAA SPECT/CT and ^90^Y PET/MR images. The contours represent registered VOIs which are delineated on CBCTs masked by the total liver. **e** Baseline MR image, with red and pink arrow pointing to the tumors which have been delineated on CBCTs (**a** and **b**). **f**, **g** Dose-volume histograms of the tumor, non-tumoral liver parenchyma, and total LPT for left and right LPT from predictive(left lobe: tumor, non-tumoral tissue, and total LPT mean dose of 73, 26, and 28 Gy; right lobe: tumor, non-tumoral tissue, and total LPT mean dose of 160, 35, and 39 Gy) and post-treatment (left lobe: tumor, non-tumoral tissue, and total LPT mean dose of 106, 22, and 27 Gy; right lobe: tumor, non-tumoral tissue, and total LPT mean dose of 119, 35, and 38 Gy) dosimetry. **h** Dose-volume histogram of the tumor, non-tumoral liver parenchyma, and total liver when combining both LPTs from predictive dosimetry (tumor, non-tumoral tissue, and total LPT mean dose of 128, 32, and 36 Gy) and post-treatment dose measurement (tumor, non-tumoral tissue, and total liver mean dose of 115, 32, and 35 Gy)

Representative images of ^99*m*^Tc-MAA-SPECT/CT, CBCTs, and post-treatment images are available in Fig. 1. One more case is provided in the supplementary material.

### Image processing

All described images in the previous section were imported into the MIM software 6.8.4 (MIM software Inc, Cleveland, Ohio) for further processing and extracted as DICOM images to be processed in IDL 8.4 (Harris Geospatial Solution, Boulder, CO, USA). All manual VOI correction/delineation has been done with MIM software, and image processing was performed using in-house software written in IDL.

#### Image registration

A multi-resolution, non-rigid registration is applied to register CBCT images to the CT from the ^99*m*^Tc-MAA study as well as the CT or MR from the ^90^Y-PET images. This algorithm is described in detail in our previous publication [8]. In short, this algorithm represents the deformation with a displacement vector in every voxel, assuming non-linear springs connect neighboring voxel pairs. Voxels are assigned to tissue classes (i.e., air, liver, non-liver tissue, and bone), and the features of the springs (i.e., stiffness, maximum deformation) can have different values for different classes. A lower rigidity is applied to the non-liver voxels compared to a high rigidity to the liver, in order to favor realistic deformations, and discourage excessive deformations in (almost) uniform regions that have hardly any features to guide the registration.

#### Liver segmentation

First, a convolutional neural network model is used to segment the entire liver on the CT image from the ^99*m*^Tc-MAA study. The convolutional neural network model is a modified version of the dual pathway, 11-layer deep, three-dimensional structure (named DeepMedic) designed for the task of brain lesion segmentation [12]. The modified model contains three pathways. Every pathway has 10 layers connected via 3×3×3 convolutional kernels followed by two common pathways based on 1×1×1 convolutional kernels. It was trained with 139 datasets from three liver segmentation challenges (SLIVER07 [13], LiTS17 [14], and Medical Segmentation Decathlon [15]) and 12 SIRT patient datasets from UZ Leuven [16]. Then, the output of the convolutional neural network (whole liver volume) was reviewed/corrected using MIM.

To segment the liver within post-treatment PET space, the deformation derived from the registration algorithm is used to transform the liver segmentation on the ^99*m*^Tc-MAA to the post-treatment space. Then, contours were exported as DICOM RTstruct sets and imported to the MIM software. A slight manual adaptation was performed using MIM software tools to compensate for imperfections as a result of the registration process and volumetric variations between post-treatment MR or CT and CT images from ^99*m*^Tc-MAA study.

#### LPT and tumor segmentation

CBCT images were analyzed by an experienced nuclear medicine physician (*CMD*) using MIM; each LPT was delineated semi-automatically using the corresponding contrast-enhanced CBCT set. To separate different LPTs, an expert drew a few lines in different transverse slices based on the contrast-enhancement in the early or late arterial phase of the CBCT, then a surface was fitted to these lines. Besides, tumors bigger than 5 ml were delineated manually on each CBCT by delineating the contrast-enhancing part of the tumor. Contours were exported as RTstruct sets and imported to the in-house software written in IDL. All VOIs (LPTs and tumors) from CBCTs were transferred non-rigidly to the pre- and post-treatment images based on the information provided by the non-rigid registration mentioned above.

For each tumor, additional steps were designed to avoid (1) a volumetric discrepancy between the original CBCT tumor segmentation and registered tumors on ^99*m*^Tc-MAA-SPECT and ^90^Y-PET space and (2) small shifts between activity map and tumor due to inaccuracies of the non-rigid registration:

- Step 1, initial alignment: using the displacement of the tumor mass centers provided by the non-rigid registration, the tumor volume was rigidly propagated on the SPECT and PET from pre- and post-treatment images.
- Step 2, location verification: the nuclear medicine expert corrected the center of each registered segmented tumor by specifying a different point with a mouse click, if that was necessary.
- Step 3, location optimization: an algorithm was designed to optimize the tumor location by maximizing the tumor uptake while minimizing the distance from the location obtained in “step 2.” For that purpose, the rigid alignment of the tumor was optimized with Powell’s algorithm. The cost function penalized large translations and prevented translation that would put the center of shifted VOI outside the boundary of the original VOI. After finding the optimum of the cost function, the final VOI was obtained by thresholding the fuzzy VOI produced by the optimization procedure.

$${\begin{aligned}cost= \left\{\begin{array}{ll} -\frac{\sum_{i \in T_{\text{new}}} A_{i} - \sum_{i \in T_{\text{init}}} A_{i} }{\sum_{i \in T_{\text{init}}} A_{i}} + \beta \times \left(\frac{\left\| \mathrm{C}_{\text{new}} - \mathrm{C}_{\text{init}} \right\|} {5 \times \mathrm{d}} \right)^{3} &, \mathrm{C}_{\text{init}} \in T_{\text{new}} \\ + \infty &, \text{otherwise} \end{array}\right. \end{aligned}} $$

Here, *T*_new_ and *T*_init_ denote the new and initial tumor VOI, *A* denotes the activity map (SPECT or PET), C_new_ and C_init_ indicate the new center point location, and the one obtained in “step 2,” and *d* denotes the diagonal of the voxel in mm (i.e., in SPECT or PET voxel size).

### Absorbed dose calculation

To perform dosimetry, we assumed: (1) there was no biological clearance, (2) the activity was exclusively injected into the planned LPTs, and (3) the energy of the activity within each voxel was fully deposited in the same voxel (local energy deposition model). To recover the total administered activity for post-treatment dose assessment, the relative post-treatment PET uptake value was used by assuming that all the administered ^90^Y activity has ended up in the liver (i.e., an LSF of 0% was used). Measuring the residual activity in treatment session is not implemented in our clinical routine. We assume that all the prepared activity is delivered to the patient, unless the interventional radiologist suspects a possible problem with the activity administration. We measured the residual activity in the vial for 10 previous administrations and these only showed a negligible amount.

In the pre-treatment assessment, to calculate the voxel-level fractional uptake, the SPECT image was normalized to the prescribed activity for each LPT to recover the LPT administered activity. This approach was taken because the portion of the administered ^99*m*^Tc-MAA within each branch prior to the pre-treatment work-up does not necessarily mimic the administered 90Y-microspheres in the treatment session.

Afterward, a map of absorbed dose in Gy was determined using the local deposition model by employing a conversion factor of:

$$\begin{aligned} \mathrm{S_{\mathrm{self-irradiation}}\left[Gy/Bq \right]}= \frac{T_{\mathrm{1/2}}[sec]}{\ln{2}} \times \frac{E_{\text{ave}} [J]} {\rho \left[kg/m^{3}\right] \times \text{vox}_{\text{vol}} \left[m^{3}\right]} \end{aligned} $$

where *T*_1/2_ is the physical half-life of ^90^Y (64.1×60^2^ s), *E*_ave_ is the average energy released per decay of ^90^Y (1.498×10^−13^ J), *ρ* is the liver tissue density (1.04×10^3^*kg*/*m*^3^), and vox_vol_ is the volume of the activity voxels in *m*^3^ which results in a conversion factor of 0.4336×10^−3^ [Gy/Bq] for ^99*m*^Tc-MAA-SPECT voxels and 2.3061×10^−3^ and 1.7607×10^−3^ for ^90^Y-PET voxels form post-treatment PET/CT and PET/MR studies, respectively [17].

### Pre- and post-treatment dosimetry comparison

Dose-volume histograms (DVHs) for all VOIs were obtained, and also, the mean dose to each VOI was computed. For each liver compartment, a series of clinically relevant dosimetry parameters were compared for predicted and post-treatment measured dose maps:

- VOI mean dose
- Dn: *n*th percentile dose, i.e., *n*% of the volume received a dose of Dn or more (D50 and D70 for TV and D30 and D50 for NTV and total LPT compartments),
- Vd: volume percentage that receives at least *d* Gy (V40 and V50 for NTV and total liver/LPT and V70 and V100 for TV compartments)

### Comparing predicted and measured doses for fixed-dose criteria

In clinical routine, the desired absorbed dose to the tumor is derived from a tumor control probability curve. Using this curve, a minimum threshold of 70 to 100 Gy is widely accepted as a dose with high tumor response probability [3, 5, 18]. So, if the ^99*m*^Tc-MAA simulation underestimates the tumor dose, but yet the tumor dose is more than these dose thresholds, this underestimation could be less critical than the opposite situation where the pre-treatment simulation suggests a mean dose of more than tumor control threshold, while the post-treatment measured dose is less than the threshold. Using this idea, a scatter plot of predicted and measured tumor dose was partitioned into four areas using 70 Gy as a dose with intermediate tumor response probability. An absorbed dose of 100 Gy is also used to illustrate a high tumor control probability:

1. The derived tumor mean dose for both predictive and post-treatment dose assessments was above 70 Gy: predictive dosimetry suggested a good tumor coverage, which was verified after treatment.
2. Both predicted and post-treatment measured tumor mean dose were below 70 Gy: predictive tumor dosimetry correctly gave an under-treatment warning.
3. Predicted tumor dose was less than 70 Gy, while the measured tumor mean dose reached the 70 Gy criterion: the result of the treatment was better than suggested by the dose prediction.
4. Predicted tumor dose was above 70 Gy while measured tumor mean dose did not reach 70 Gy: ^99*m*^Tc-MAA dosimetry falsely predicted a good tumor coverage which did not materialize after treatment.

The same approach was applied for non-tumoral tissue mean dose using normal tissue complication probability curves. A dose of 50 Gy is considered as a safety threshold. An absorbed dose of 40 Gy is the maximal recommended dose in case of cirrhotic non-tumoral liver tissue or patient heavily treated with chemotherapy [3, 5]:

1. The derived NTV mean dose for both predictive dosimetry and dose measurement was below 50 Gy: predictive dosimetry could predict the safety of the treatment for non-tumoral liver parenchyma.
2. Both predicted and post-treatment measured NTV mean dose was above 50 Gy: predictive NTV dosimetry correctly identified a dose range with a non-zero risk for liver complications.
3. Predicted NTV dose was above 50 Gy, while post-treatment measured mean dose to this VOI did not reach 50 Gy: predictive dosimetry falsely suggested a potential risk for liver toxicity.
4. Pre-treatment NTV dose was less than 50 Gy, while measured mean dose of NTV exceeded the 50 Gy limit: pre-treatment dose prediction did underestimate the risk for liver toxicity.

### Comparing predicted and measured doses to the planned dose

For patients whose activity planning was done by employing the partition model, the therapy team agreed on a delivered dose to the non-tumoral liver and tumor compartment, which are called “planned dose criteria” in this document. To calculate the injected activity, the total liver and liver perfusion territories have been drawn on baseline images and tumors have been delineated by thresholding the ^99*m*^Tc-MAA uptake.

The planned dose to the NTV and TV was compared to the doses obtained from predictive dosimetry and post-treatment dose measurement using the relative difference between calculated dose and planned dose in percentage. For predictive dosimetry, deviation from zero indicates the effects of the different VOI definition method used in this study (perfusion based LPT and tumor segmentation on CBCTs) compared to activity planning VOI definition (using anatomical landmarks on CT or MR for LPT segmentation and thresholding ^99*m*^Tc-MAA uptake for tumor definition). Considering post-treatment dose measurement, the difference between planned dose and determined mean dose could be caused by a difference in VOI definition or variation between ^99*m*^Tc-MAA and therapeutic microspheres distribution. The differences are calculated in a way that negative values show doses below and above the planned dose for NTV and TV compartments, respectively.

### Statistical analysis

R software version 3.6.1 (R Foundation for statistical computing, Vienna, Austria) was used for all statistical analysis. A Passing–Bablok regression scatter plot was used to compare dose parameters from predictive and post-treatment dose assessment by displaying the regression line and confidence intervals. This method is commonly used to compare two measurement methods by plotting them in *x*- and *y*-axis. Also, Pearson correlation was used to evaluate the agreements; *r* values greater than 0.3, 0.5, and 0.7 were considered as a weak, moderate, and strong positive linear relationship. In Passing–Bablok graphs, the identity line is displayed using a dashed line, and the solid line and shaded area represent the regression line and 95% confidence interval.

A Bland–Altman plot was also used to calculate the agreement between predicted and post-treatment measured dose parameters. In the Bland–Altman plots, the middle dashed line represents the mean difference, and the purple area is the confidence interval; the green and pink area and their dashed lines also showed the difference ± standard deviation and their confidence intervals to give a visual impression of the precision of these parameters.

Dose parameters for predictive dose and post-treatment dose measurement were also compared using paired Wilcoxon rank-sum test. A *p* value of less than 0.05 was set as a significance threshold.

## Results

### Patient and treatment characteristics

**Fig. 2:**
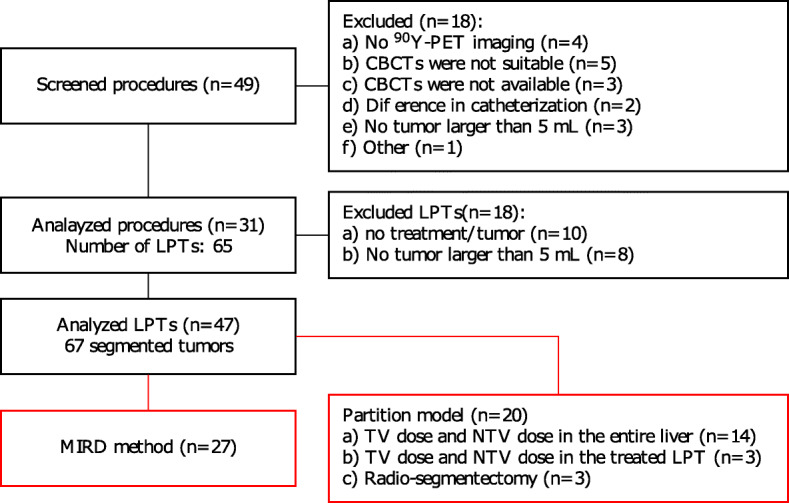
Flowchart of patient selection (consort diagram in black blocks) and activity planning in the selected LPTs (red blocks)

Of 49 consecutive patients identified, 31 patients were included, and 18 patients were excluded (see Fig. 2): in 4 patients, the post-treatment PET image was not acquired, only bremsstrahlung SPECT images were available; in 5 patients, the quality of CBCT information was not suitable for tumor and/or LPT delineation; in 3 patients, CBCT images were not available; in 2 patients, the treatment team decided to change the therapy strategy and put the catheter in a different position for the therapeutical procedure compared to the ^99*m*^Tc-MAA work-up; and in 3 patients, all tumors were smaller than 5 ml and were not considered for dose assessment as partial volume effects are too pronounced. Finally, in the last excluded patient, some voxels within the liver received blood from both administrations within different catheter tip positions in pre-treatment work-up. So, it was not possible to extract fractional uptake for each administration from the ^99*m*^Tc-MAA-SPECT image.

Of those 31 patients included, 67 tumors (with a volume bigger than 5 ml) in 47 LPTs (from a total of 65 defined LPT) were included in this retrospective study. The baseline characteristics for the patients and treatment sessions (LPT) are described in Table 1.

**Table 1:** Patient characteristics

Patient characteristics	
Sex (female/male)	12/19
Age in years, median [range]	67 [25–83]
Height in meter, median [range]	1.70 [1.52–1.83]
Weight in kg, median [range]	74 [48–116]
Tumor type, *n* (%)	
HCC	19 (61.3%)
No cirrhosis	5
Proven cirrhosis	14
NASH	7
ASH	4
HBV	1
HCV	1
Unknown etiology	4
CRC	6 (19.4%)
NET	3 (9.7%)
Pancreas	1 (3.2%)
Breast	1 (3.2%)
Stomach	1 (3.2%)
Portal vein embolism, *n*	
Patients with PVE	2
Prior treatment, *n* (median months between last cycle and SIRT [min,max])	
Systemic treatment	13 (1.0 [0.3, 15.2])
Anti-angiogenic ⋆	6 (9.7 [0.5, 16.6])
Anti-angiogenic (directly before SIRT)	3 (0.9 [0.5, 15.2])
Anti-angiogenic (not directly before SIRT)	3 (14.1 [5.3, 16.6])
Non anti-angiogenic ⋆⋆	7 (0.7 [0.3, 13.4])
RFA/MWA	6 (13.8 [3.5, 31.0])
TACE	4 (8.3 [4.1, 48.3])
Resection	3 (14.4 [1.3, 41.9])
Volumes from prescription sheet (ml), median [range]	
Total liver	1741 [789–4122]
Non-tumoral liver	1538 [682–2942]
Tumor	134 [0–2785]
Tumor burden	
Median [range], %	8.8 [0.0–67.6]
< 5%, *n* (%)	10 (32%)
5–10%, *n* (%)	8 (26%)
10–25%, *n* (%)	8 (26%)
25-50%,n (%)	3 (10%)
> 50%,*n* (%)	2 (6%)
MAA work-up information	
Lung shunt fraction (%)	8.0 [0.0–13.9]
Time from treatment in days, median [range]	18 [9–46]
Prescription method	
Non-compartmental partition model, *n* (%)	16 (52%)
Compartmental partition model, *n* (%)	14 (45%)
Mixture of models, *n* (%)	1 (3%)
Treatment strategy	
Whole liver, *n* (%)	2 (6%)
Bi-lobar, *n* (%)	15 (48%)
Mono-lobar, *n* (%)	10 (32%)
Selective (3 segments), *n* (%)	3 (10%)
Selective (4 segments), *n* (%)	1 (3%)
Prescribed activity	
Total administered activity (GBq) [range]	1.527 [0.383–3.700]
Number of analyzed tumors (bigger than 5 ml)	
Patients with 1 tumor, *n* (%)	16 (52%)
Patients with 2–3 tumors, *n* (%)	11 (35%)
Patients with more than 3 tumors, *n* (%)	4 (13%)
Analyzed LPTs information from prescription sheet	
Total LPT (cc), median [range]	1001 [57–3172]
Non-tumoral liver (cc), median [range]	849 [0–2268]
Tumor (cc), median [range]	67 [0, 2785]
LPT to total liver ratio (%), median [range]	54 [3–100]
Tumor burden	
Median [range], %	9.4 [0.0–100.0]
< 5%, *n* (%)	16 (34%)
5–10%, *n* (%)	9 (19%)
10–25%, *n* (%)	11 (23%)
25–50%, *n* (%)	8 (17%)
> 50%, *n* (%)	3 (6%)
Post-treatment image modality	
PET/MR - PET/CT, *n*(%)	28 (90%) – 3 (10%)

^⋆^Aflibercept, Regorafenib, Bevacuzimab, or Sorafenib

^⋆⋆^Octreotide LAR, FOLFOX, FOLFIRI, Cetuximab, De Gramont, TAS102, Avelumab, Paclitaxel, epirubicin + cisplatin + fluorouracil, Exemestane, Gemcitabine + abraxane, Everolimus, Letrozole, Trastuzumab, Pertuzumab, Panitumumab, Lanreotide

### Activity planning

As detailed in Fig. 2, of those 47 LPTs, the non-compartmental partition model (MIRD model) was used for 15, 11, and one LPT(s) aiming at 40, 50, and 60 Gy, respectively. For the rest, a compartmental partition model was used with a personalized tumor dose criterion and/or a tissue sparing criterion. In this model, a personalized tumor dose criterion (95 Gy, *n* = 1 ; 120 Gy, *n* = 4; 125 Gy, *n* = 1; 135 Gy, *n* = 1; 150 Gy, *n* = 5; 160 Gy, *n* = 1; 240 Gy, *n* = 2; 250 Gy, *n* = 2; 300 Gy, *n* = 3) was used. This model also aimed at sparing liver tissue parenchyma from a certain absorbed dose (non-tumoral liver parenchyma of the entire liver dose of 15 Gy, *n* = 2; 20 Gy, *n* = 1; 25 Gy, *n* = 7, and 30 Gy, *n* = 4, or non-tumoral liver parenchyma of the LPT dose of 20 Gy, 30 Gy, and 40 Gy each for one LPT, and radio-segmentectomy for 3 LPTs).

More details are provided in Table 1.

### VOI segmentation

Considering the 31 patients, there was a high correlation between total liver volumes (Pearson *r* = 0.990) determined on post- and pre-treatment studies; the ratio of the volumes had a median of 1.01 with a first and third interquartile range of 0.99 and 1.04. These volume differences represented either the volume change during the registration or a real biological change.

For 47 analyzed LPTs, the ratio between the volumes defined on post- and pre-treatment images had a median of 1.03 with a first and third interquartile range of 0.98 and 1.07. Again, rather than possible volume change associated with the non-rigid registration, this difference could be explained by a possible biological change.

The LPT to whole-liver volume ratio is a commonly used parameter in SIRT dosimetry. In some prescription models (e.g., BSA and MIRD model), this parameter is used to divide the total prescribed vial to be administrated in different branches of the hepatic artery. When comparing this parameter for LPTs defined on CBCTs with the numbers reported on the prescription sheet, the ratio had a median of 0.99 with a wide interquartile range (first and third interquartile range 0.90 and 1.09 respectively) which can directly affect the dosimetric analysis.

Two more examples are provided in the supplementary material: (1) an LPT segmentation example on CBCTs, which could be considered superior to a classical liver lobe/segment delineation because of a specific catheterization and (2) an example of lobar treatment (with bilobar work-up) which shows that CBCT-based LPT segmentation nicely followed the activity distribution of the yttrium-90.

### Predicted and measured doses

**Fig. 3:**
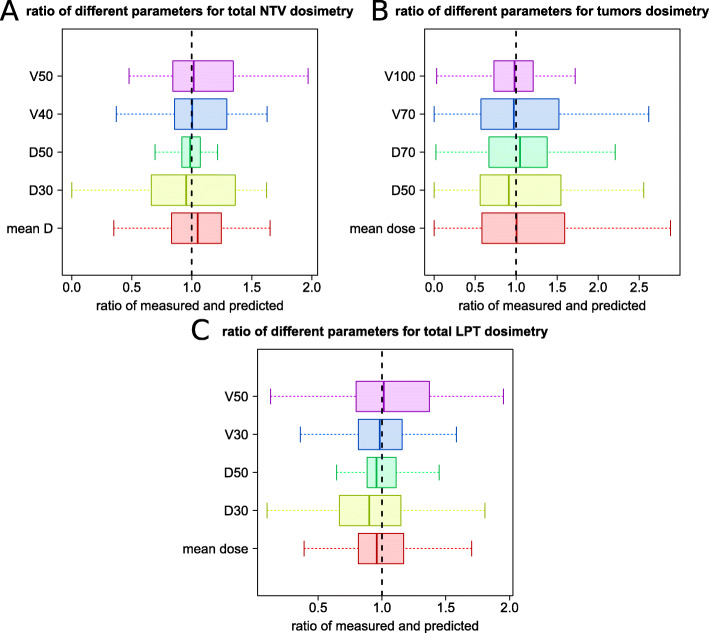
Box plot of the ratio of different dosimetry parameters derived from **a** NTV in the entire liver, **b** total LPT (TV and NTV together), and **c** tumors (the outliers were excluded for visual purposes)

Absorbed dose distribution parameters of each LPT derived from pre-treatment ^99*m*^Tc-MAA-SPECT and post-treatment ^90^Y-PET images were compared for total liver NTV, total LPT, and tumors. Table 2 and Fig. 3 summarize the main dosimetric comparison between predictive and post-treatment dosimetry without considering the outliers. The following sections provide more details about this comparison.

**Table 2:** Statistical properties of different dosimetric parameters in different VOIs

		Predicted		Post-treatment		Wilcoxon	Pearson correlation	
VOI	Parameter	Mean	std	Mean	std	*p* value	*r*	*p* value
Total NTV	Mean dose	30.4	11.9	29.5	11.4	0.272	0.937	**<0.001**
	D30	31.4	15.8	32.3	17.0	0.344	0.826	**<0.001**
	D50	18.6	11.2	17.7	12.3	0.355	0.803	**<0.001**
	V40	24.5	11.1	25.1	12.3	0.666	0.805	**<0.001**
	V50	17.6	7.9	18.7	9.8	0.249	0.826	**<0.001**
Total LPT	Mean dose	63.5	85.6	53.0	52.4	0.135	0.820	**<0.001**
	D30	68.4	95.2	56.8	61.0	0.440	0.755	**<0.001**
	D50	41.8	60.6	31.6	31.7	0.076	0.756	**<0.001**
	V30	48.6	19.9	45.8	17.4	0.286	0.732	**<0.001**
	V50	31.0	19.6	29.6	16.2	0.641	0.772	**<0.001**
Tumors	Mean dose	152.3	144.7	143.0	137.8	0.918	0.623	**<0.001**
	D50	139.2	129.8	127.8	134.7	0.576	0.597	**<0.001**
	D70	102.8	95.3	87.9	98.6	0.080	0.604	**<0.001**
	V70	64.9	35.4	60.4	30.1	0.275	0.229	0.063
	V100	52.5	36.1	47.4	30.3	0.354	0.381	**<0.001**

Two examples of TV, NTV, and total LPT dosimetry are shown in Figs. S1 and 1.

#### Total liver non-tumor volume

A summary of the tumor dose comparison between ^99*m*^Tc-MAA and ^90^Y distribution has been shown in Table 2 and Fig. 3. The Wilcoxon test did not show any significant difference in any of the dosimetry parameters from the total liver NTV compartment. The mean dose to the total non-tumoral tissue was 30±12 and 30±11 Gy in predictive dosimetry and post-treatment dose measurement; the ratio of measured and predicted mean doses have a median of 1.05 (first and third interquartile range of 0.83 and 1.25). Both predicted and measured dosimetry showed that on average, only around 25% of the non-tumoral tissue parenchyma received more than 40 Gy; the ratio of measured and predicted V40 had a median of 1.00 (first and third interquartile range of 0.86 and 1.29). These volumes were 18% and 19% for predictive dosimetry and post-treatment dose measurement for 50 Gy threshold level; the ratio of measured and predicted V50 had a median of 1.02 (first and third interquartile range of 0.84 and 1.35). The D30 values showed that only 30% of the liver received more than 31 and 32 Gy using predicted and measured doses; the ratio of measured and predicted D30 had a median of 0.95 (first and third interquartile range of 0.66 and 1.36).

**Fig. 4:**
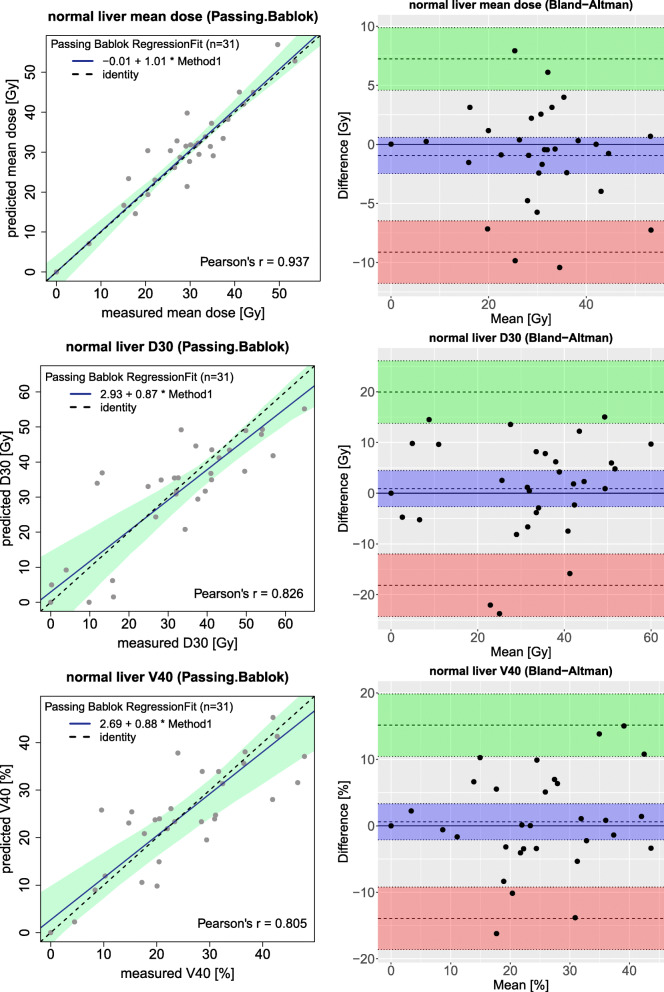
Passing–Bablok (left figures) and Bland–Altman (right figures) plots for different dosimetry parameters derived from NTV in the entire liver; mean dose to the non-tumoral liver in the first row, non-tumoral liver D30 in the middle row, and non-tumoral liver V40 in the last row

A Passing–Bablok regression, which has been shown in Fig. 4, estimated a strong correlation of the mean dose to the non-tumoral liver between predicted and measured doses (*r* = 0.937). Other reported dose parameters for NTV (D30, D50, V40, and V50) also showed a moderate correlation between ^99*m*^Tc-MAA and ^90^Y-PET based dosimetry (*r* bigger than 0.750 for all).

Bland–Altman analysis of NTV gave a mean difference of − 0.9 Gy (− 9.1, 7.2) for mean dose, 0.9 Gy (− 18.2, 19.9) for D30, and − 0.6% (− 13.9, 15.1) for V40. The difference between predicted and measured mean dose to the total NTV doses did not exceed 11 Gy.

#### LPT volumes

Table 2 and Fig. 3 provide some information about the dose to the total LPT comparison between ^99*m*^Tc-MAA and ^90^Y distribution. The mean dose of the total LPT estimated on ^99*m*^Tc-MAA and post-treatment dose measurement were 63.5 and 53.0 Gy. The V30 for pre-treatment dose prediction and post-treatment dose calculations were 49±20 and 46±17 %. For other dosimetric parameters, no significant difference was observed.

**Fig. 5:**
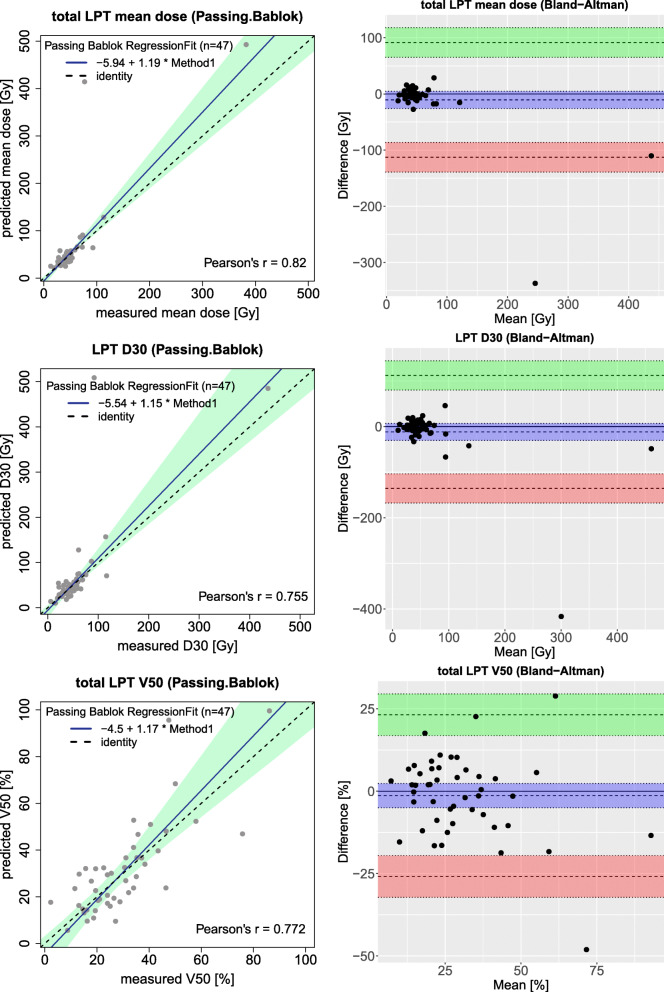
Passing–Bablok (left figures) and Bland–Altman (right figures) plots for different dosimetry parameters derived from total LPT (TV and NTV together); mean dose to the total LPTs in the first row, total LPT D30 in the middle row, and total LPT V50 in the last row

Bland–Altman analysis of LPT (see Fig. 5) gave a mean difference of − 10.5 Gy (− 112.7, 91.6) for mean dose, − 11.7 Gy (− 135.7, 112.4) for D30, and − 1.3% (− 25.9, 23.2) for V50. The difference between predicted and measured mean dose to the LPT doses did not exceed 25 Gy except for two outliers (i.e., radio-segmentectomy strategy).

#### Tumor volumes

In Table 2, a summary of the tumor dose comparison based on the ^99*m*^Tc-MAA-SPECT and ^90^Y-PET distributions is shown. Predicted and measured dosimetry showed an average of 152 and 143 Gy mean dose to the tumor. As illustrated in Fig. 3, the median of the ratio between measured and predicted tumor mean dose was 1.01, but a larger deviation was reported for predictive and measured dose to the tumors compared to the LPT and NTV. The first and third interquartile range of the ratio of the mean doses were 0.58 and 1.59, which reflected a considerable discrepancy between measured and predicted dose to the tumor volumes.

On average, half of the tumors’ volume received an absorbed dose of 139 and 128 Gy or more based on predictive dosimetry and post-treatment dose measurement; the ratio of measured and pre-treatment predicted D50 has a median of 0.91 (first and third interquartile range of 0.56 and 1.55). Based on analyzing V70, around 65% and 60% of the tumor volumes received more than 70 Gy on predictive and measured dosimetry; these volume portions are reported around 53% and 47% for 100 Gy dose threshold level for predictive and post-treatment dose assessments. Seventy percent of tumor volumes received at least 103±95 Gy using predictive dosimetry while post-treatment dose calculation showed that 70% of the tumor volumes received at least 88±99 Gy; the median, first, and third interquartile ranges of the ratio between D70 in post-treatment dose measurement and predictive dosimetry were 1.05, 0.67, and 1.38 respectively.

**Fig. 6:**
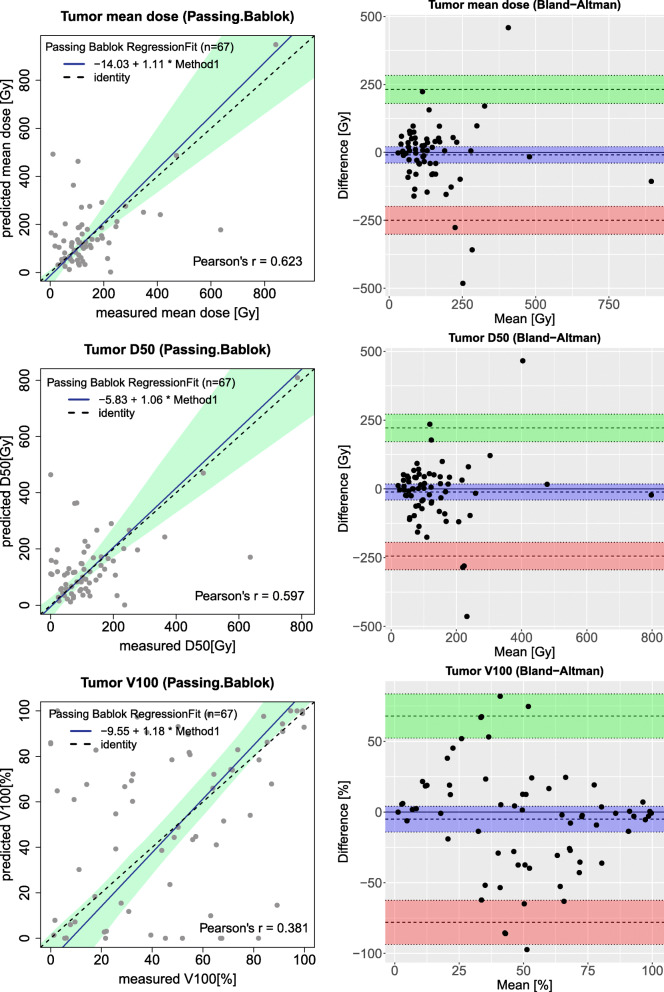
Passing–Bablok (left figures) and Bland–Altman (right figures) plots for different dosimetry parameters derived from tumors; mean dose to the tumors in the first row, total tumors D50 in the middle row, and tumors V100 in the last row

A Passing–Bablok and Bland–Altman analysis of mean dose to the tumor and D50 and V100 is shown in Fig. 6 that shows remarkable higher variations and relatively limited agreement between predicted and measured dose compared to NTV and LPT. Moderate correlations existed between the mean dose of pre-treatment simulation and post-treatment dose measurement (*r* = 0.623). On the other hand, our data does not show a significant correlation between other tumor parameters derived from DVH. Bland–Altman analysis of tumors gave a mean difference of − 9.3 Gy (− 249.9, 231.3) for the mean dose.

### Comparing predicted and measured doses for fixed dose criteria

In the previous section, pre-treatment dose simulation and post-treatment dose measurement in different VOIs have been compared using mean dose and different DVH parameters.

**Fig. 7:**
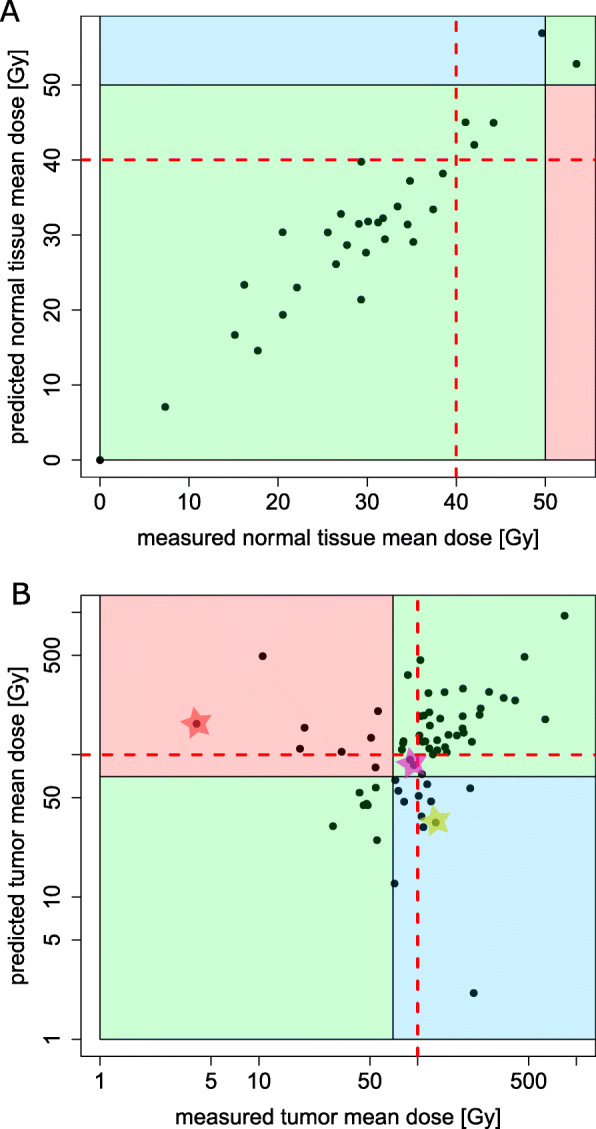
Scatter plot with clinically relevant dose intervals in **a** NTV and **b** TV VOIs; lower green area: where tumor/non-tumoral liver dose from both predictive and post-treatment dosimetry is less than critical dose; upper green area: where tumor/non-tumoral liver dose from both predictive and post-treatment dose assessment is more than critical dose; blue area: where tumor/non-tumoral liver dose is bigger than critical dose from measured/predictive treatment dosimetry but less than defined dose from predicted/measured doses; red area: where tumor/non-tumoral liver dose is bigger than critical dose from predicted/measured doses but less than defined dose from measured/predicted doses; the red, pink, and yellow stars correspond to the tumors which have been described in the Fig. 8 (patient ID 20). The red dashed lines correspond to the lowest dose recommended for non-tumoral tissue and the high tumor control probability for **a** and **b**, respectively

Figure 7 represents a scatter plot that shows predicted versus measured mean dose with four colored areas: the green area is the area in which both predicted and measured doses are either less than 50 Gy or more than 50 Gy (*n* = 29 and 1 from 31 patients); the blue area represents the patients whose pre-treatment simulation estimates the NTV mean dose of more than 50 Gy, while ^90^Y-microsphere distribution showed a dose of less than 50 Gy after treatment (*n* = 1); the red area is the risky area where ^99*m*^Tc-MAA simulates a safe treatment, whereas the actual dose to a NTV was more than 50 Gy (*n* = 0). More details are provided in Table 3.

**Table 3:** Comparing predicted and measured TV doses and fixed dose criteria; joint- histogram of tumor absorbed dose using bins of 0, 70, and 100 Gy that corresponds to no tumor irradiation, intermediate, and high tumor control probability, respectively

		Measured TV dose			
		Low dose	Intermediate dose	High dose	Total
		[0, 70] Gy	[70, 100] Gy	[100, +*∞*] Gy	
Predicted TV dose	High				43
	[100, +*∞*] Gy	7	4	32	
	Intermediate dose				4
	[70, 100] Gy	1	2	1	
	Low dose				20
	[0, 70] Gy	7	5	8	
	Total	15	11	41	67

**Fig. 8:**
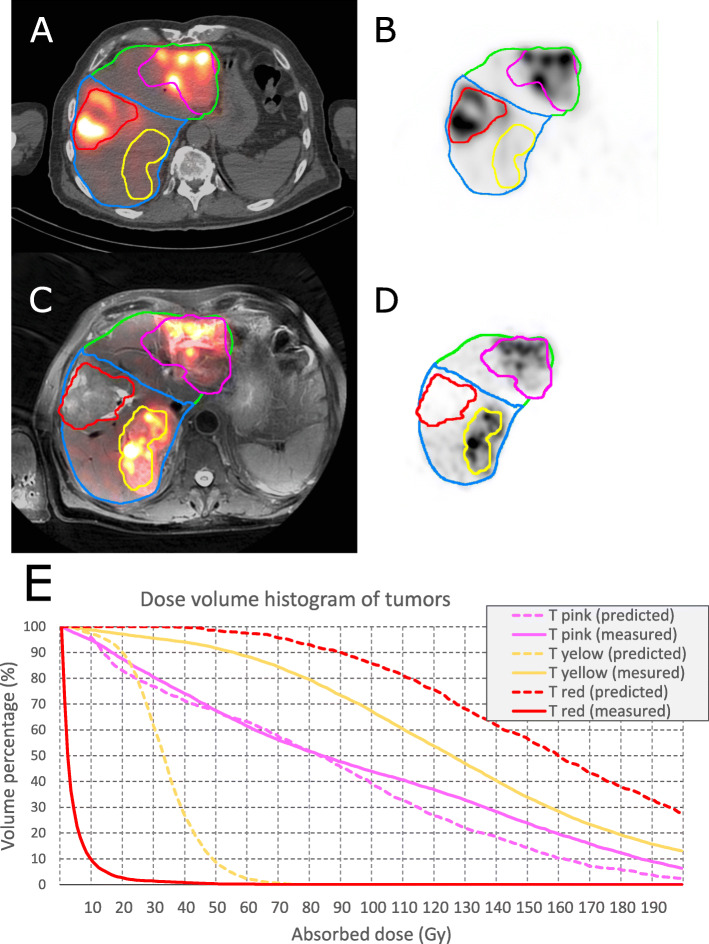
An example (patient ID 20) of disagreement between tumor dose between predictive and post-treatment dosimetry. **a**, **b** Pre-treatment image (^99m^Tc-MAA-SPECT/CT). **c**, **d** Post-treatment image (^90^Y-PET/MR); the tumor contoured in pink: predictive and post-treatment dose assessment were comparable (pre-treatment predicted: mean dose: 84 Gy, D50 = 83 Gy and V100 = 39%; post-treatment: mean dose: 95 Gy, D50 = 84 Gy and V100 = 44%); the tumor contoured in yellow: predictive dosimetry underestimated the tumor dose (pre-treatment predicted: mean dose: 33 Gy, D50 = 33 Gy and V100 = 0%; post-treatment measurement: mean dose: 130 Gy, D50 = 125 Gy and V100 = 67%); and the tumor contoured in red: predictive dosimetry over-estimated the tumor dose (pre-treatment predicted: mean dose: 165 Gy, D50 = 159 Gy and V100 = 86%; post-treatment: mean dose: 4 Gy, D50 = 2 Gy and V100 = 0%). **e** Dose-volume histogram of the described VOIs based on predictive and post-treatment dose assessments. In this case a different catheter positioning between MAA work-up and treatment resulted in flow variation. A preferential targeting of the tumor in the ventral part of the right liver lobe (red tumor) was observed in the work-up. On the other hand, after ^90^Y-microsphere injection, a preferential targeting of the tumor in the dorsal part of the left liver lobe was obtained. This is one example of a potential pitfall in SIRT in general and a cause of discrepancy between ^99m^Tc-MAA and ^90^Y-PET in particular

The same plot is provided in Fig. 7 for TV compartments. For 39 out of all 67 tumors, both predictive and post-treatment dose assessment suggests a tumor mean dose of more than 70 Gy (upper green area); for seven tumors, measured tumor dose was less than 70 Gy, and ^99*m*^Tc-MAA simulation indicated this as well (lower green area); the blue area is the area where predictive dosimetry failed to estimate a dose of more than 70 Gy, while the actual treatment reached that dose limit (*n* = 13); for eight tumors, pre-treatment simulation suggested a sufficient dose (more than 70 Gy) and post-treatment dose measurement revealed that the microsphere treatment did not meet this limit; the prediction was too optimistic here. Figure 8 shows an example of this mismatch between predictive dosimetry and post-treatment dose measurement. Table 4 provides more details.

**Table 4:** Comparing predicted and measured NTV doses and fixed dose criteria; joint- histogram of total NTV absorbed dose using bins of 0, 40, and 50 Gy that corresponds to no non-tumoral irradiation, lowest dose recommended to the non-tumoral compartment, and safety threshold, respectively

		Measured NTV dose			
		Low dose	Intermediate dose	High dose	Total
		[0, 40] Gy	[40, 50] Gy	[50, +*∞*] Gy	
Predicted NTV dose	High				2
	[50, +*∞*] Gy	0	1	1	
	Intermediate dose				3
	[40, 50] Gy	0	3	0	
	Low dose				26
	[0, 40] Gy	26	0	0	
	Total	26	4	1	31

### Comparing predicted and measured doses to the planned dose

**Fig. 9:**
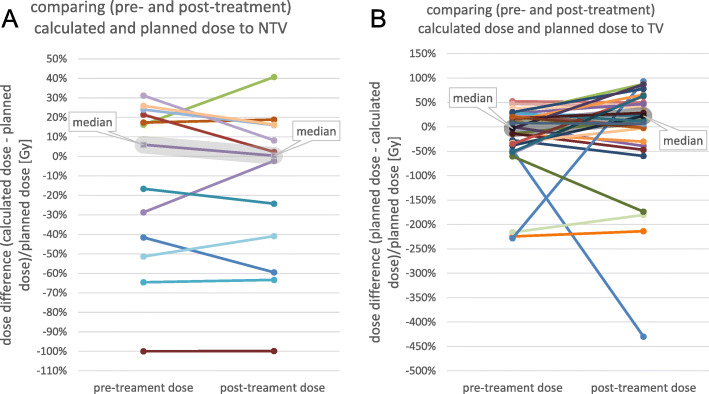
A comparison between calculated doses, and prescribed doses to the **a** NTV and **b** TV; the difference between obtained dose and prescribed dose is provided in a way that for tumor graph negative values correspond to doses above prescribed dose and for non-tumoral tissue graph it is in the opposite way

For 13 patients, the injected activity was prescribed by applying a compartmental partition model by thresholding ^99*m*^Tc-MAA uptake to distinguish between TV and NTV. Figure 9 provides the relative difference between calculated absorbed dose derived from predictive and post-treatment analysis of NTV and the planned absorbed dose. So, negative values correspond to a lower dose to the NTV than what has been prescribed, which has happened in 6 patients for both predictive dosimetry and post-treatment dose measurement. The median (first, third interquartile) for this relative difference was 6% (− 42, 21%) and 0% Gy (− 41, 16%) for predictive dosimetry and post-treatment dose measurement.

For these 13 patients for whom the partition model was used for prescription, measured and predicted absorbed doses of 30 tumors were compared with TV planned dose. Figure 9 represents the relative difference between the planned dose to the TV and the calculated dose. The negative values correspond to the cases where the calculated tumor dose is greater than what has been prescribed, which has been reported for 16 and 10 tumors for predictive dosimetry and post-treatment dose measurement, respectively. The median (first, third interquartile) of relative difference for pre-treatment predictive and post-treatment analysis was − 3% (− 38, 25%) and 22%(− 30, 62%) respectively.

## Discussion

This study presents a framework to compare predicted and measured absorbed doses for SIRT using CBCT-based VOI segmentation. To our knowledge, implementation of the contrast-enhanced CBCT in voxel-level dose comparison has previously not been studied. In other studies, either anatomical landmarks on non-catheter based anatomical imaging modalities (e.g., MR or CT) or activity uptake-based imaging modalities (PET or SPECT) were used to define LPTs corresponding to different catheter positions. We demonstrated that activity planning using ^99*m*^Tc-MAA absorbed dose distribution is related to post-treatment microsphere dose distribution in the tumor, non-tumoral, and LPT compartments. The quantitative analysis showed that the correlation between ^99*m*^Tc-MAA absorbed dose distribution is higher in the non-tumoral compartment than in the tumors. The non-tumor mean dose could be used in treatment planning to predict if the normal liver parenchyma receives a safe absorbed dose (e.g., less than 50 Gy). In our analysis, we report a correlation between predicted and measured tumor absorbed dose employing a tumor-by-tumor approach instead of the patient averaged tumor dose. Analyzing the averaged predictive and measured tumor dose may lead to artefactual good correlation between pre- and post-therapy imaging. For example, Figs. 1f and g show large deviations between predictive and measured dose for the left and right sided tumors (under- and overestimation respectively) which balance each other and result in a good correlation when the tumor absorbed doses in the entire liver are averaged (Fig. 1h). Our results show that for most of the tumors, ^99*m*^Tc-MAA-based dosimetry could correctly predict if the dose to the tumor was sufficient or insufficient (e.g., more than 70 Gy).

Predicted absorbed dose estimation is becoming more and more crucial for individual SIRT planning [3]. Bastiaannet et al. reviewed the strategies to optimize activity prescription to the current state of radiobiological knowledge regarding SIRT and the current possibilities of performing predictive dosimetry and post-therapy dose measurement [3]. The BSA method is still the most used model for activity prescription. Nevertheless, activity planning using BSA method is driven by patient’s BSA used as a surrogate of real patient’s liver volume [19], which can lead to an overestimation (previous surgery) or underestimation (hepatomegaly caused by the presence of tumor) of the real liver volume [20]. Partition model or voxel-level dosimetry is the potential alternative for prescribing the activity, but applying an accurate partition model is challenging because it relies on VOI segmentation techniques and predictive value of the ^99*m*^Tc-MAA particles.

A disagreement between predicted and measured absorbed dose parameters can occur due to several factors [3, 21]; it could reflect the actual discrepancy between ^99*m*^Tc-MAA particles and ^90^Y-microspheres, as well as the inaccuracy in imaging and/or dose calculation and reporting techniques. Besides the difference in physical characteristics of two used radionuclides for dose prediction and measurement, a different catheter positioning and administration speed, vasospasm during one of the injection session, the embolic effect of the resin microspheres, and change in tumor vascularization between pre-treatment simulation and treatment session can lead to a weak predictive power of ^99*m*^Tc-MAA particles. During the SIRT procedure, attempts were made to use the exact positioning of the catheter during the ^99*m*^Tc-MAA injection and report any mismatching between the catheter tip position. In this study, clinical reports have been reviewed carefully for all screened procedures, and in case of a reported difference in catheterization, the case was excluded from the study.

Calibrated activity in the vial is reported with an error of 20% [22], which can have a direct effect on dosimetry accuracy. Generally, ^90^Y-TOF-PET imaging is considered to be quantitative with a limited margin of error. To avoid any systematic bias between the total activity (and absorbed dose) in the liver, the activity distribution of the post-treatment ^90^Y-PET image is rescaled based on the assumption that the total PET activity in the liver should equal the total prepared activity, considering no residual activity in vial nor catheters. On the other hand, the fractional uptake obtained from ^99*m*^Tc-MAA-SPECT is rescaled assuming that the total predicted activity in each LPT should equal the administered activity within the corresponding catheter tip position.

In both predictive and measured dose calculation, the LSF estimation based on planar imaging was not used at face value in the dose calculation as it overestimates the true lung shunt measurement [23]. In our center, the patients with a real lung shunt (planar LSF > 30%; macroscopic vascular connections visible on angiography) are excluded from SIRT. In analyzed patients, the highest LSF was 13.9%. In these circumstances, we believe that the true lung shunting was close to 0%, with a marginal signal coming from the lungs due to the smallest particles in the ^99*m*^Tc-MAA solution, in vivo degradation products moving from the liver to the lungs, and scatter from the liver. This is substantiated by absence of uptake in the lungs according to ^90^Y-PET in all our patients.

Lastly, any technical aspects of the VOI definition may alter the correspondence between predicted and measured dose parameters. To minimize this inaccuracy, additional control steps have been designed in this study: (a) liver segmentation by CNN in ^99*m*^Tc-MAA-SPECT space and registered liver VOI in ^90^Y-PET space have been verified and modified by an expert. The ratio between liver volumes has a first, second, and third interquartile range of 0.99, 1.01, and 1.04, respectively. (b) For segmenting the liver perfusion territory corresponding to each catheter tip position, contrast-enhanced CBCT imaging is used. In our previous study, it has been shown that by aiming at delivering 40 Gy to the total liver and prescribing activity based on CT-based liver perfusion territories, absorbed dose to the right and left CBCT-based LPT has a median (range) of 40.8 Gy ([34.1, 48.8] Gy) and 38.1 Gy ([19.3, 49.1] Gy) [8]. (c) To optimize the delineation of the tumors, a multi-step hybrid approach (which combines anatomical and physiological information) has been used. Segmented tumors on CBCT images have been projected on activity maps using non-rigid registration. An additional verification step has been designed to avoid wrongfully assigning the tumor to a nearby non-tumoral high or low uptake area or mislabeling two tumors next to each other. By assuming a high local uptake of the tumor, a cost function was designed to shift the tumor VOI very locally to capture as much activity as possible.

We found a strong correlation between non-tumoral liver mean dose, D30, and V40. Our results for mean tumor dose confirm the moderate correlation between predicted and measured tumor dose reported by Gnesin et al. [7]. They also found a better agreement between NTV compartment than tumors. This possibly reflects the fact that a better overall relation could be achieved in larger volumes (NTVs) than smaller volumes (TVs). Recently, Jadoul et al. also reported similar results for HCC tumors [24]. In this study, for LPT segmentation, thresholding the activity uptake by 1% of the maximum activity was used.

Considering the reliability of ^99*m*^Tc-MAA absorbed dose estimation, Chiesa and Maccauro discussed the usefulness of extracting the correlation between predictive and post-therapy dosimetry parameters from a patient population to ”optimize treatment on the average” [25]. Indeed, the interpretation of a moderate correlation between predictive and measured dose in a patient-tailored treatment planning is still debatable without considering the confidence intervals. For example, a moderate correlation was established for the tumor dose but Fig. 6 illustrates many tumors with an overestimation or underestimation of the tumor dose using predictive dosimetry. As can be seen in this figure the confidence interval for tumor mean dose in the Bland–Altman figure is about 250 Gy while the confidence interval for non-tumoral mean dose is [ − 9, 7] Gy (see Fig. 4). So, an accurate tumor dose prediction from the pre-treatment data for a specific patient was not reached.

We adopted 70 Gy as the requirement for tumor response and 50 Gy as the safety threshold for non-tumor irradiation. Our results showed that for 97% of the patients, the ^99*m*^Tc-MAA mean dose could predict either a safe activity planning or over-dosing of the healthy tissue (e.g., radio-segmentectomy). Also, for 69% of the tumors, ^99*m*^Tc-MAA-based dose estimation could predict if the dose to the tumor was sufficient (more than 70 Gy).

We also compared predicted and measured absorbed doses to the projected (planned) absorbed dose. The planned absorbed dose has been calculated before treatment to prescribe activity based on the partition model. In this dosimetry scheme, activity thresholding is used to calculate tumor to normal activity concentration ratio. So, any inconsistency between planned absorbed dose and predicted absorbed dose reflects the effect of different applied VOI definition (activity thresholding and anatomical tumor segmentation) solely. Notably, segmenting the tumoral lesion into different compartments (viable tumor and necrosis) is dependent on the imaging modality used. Here, we delineated the contrast-enhancing part of the tumor on CBCT (hypervascular areas) which could be different from tumor segmentation on MR or thresholding the ^99*m*^Tc-MAA-SPECT or ^18^F-FDG-PET. Taking necrosis into account is more important for tumors with a high percentage of necrosis, typically large tumors. Our analysis suggests a median (first and third interquartile range) difference of 6% ([ − 42, 21]%) and − 3% ([ − 38, 25]%) between the projected and predicted mean dose for the non-tumoral and tumor compartments, respectively. It confirms that thresholding the ^99*m*^Tc-MAA could result in an overestimation of the tumor dose prediction up to 50%. A more substantial discrepancy has been observed when comparing the planned dose and measured doses.

Several limitations could be mentioned for this study. First, the study design is retrospective. Also, the number of analyzed cases was limited; further studies, including more patients in homogeneous tumor types, are required to determine a better evaluation of the predictive value of ^99*m*^Tc-MAA. In addition, detailed lesion analysis (e.g., separating necrosis and viable tumor) could be performed in the future to refine tumor dosimetry.

## Conclusion

In this study, we demonstrated that CBCT-based dose estimation using a ^99*m*^Tc-MAA study is related to the post-treatment dose measurement. The agreement is stronger for non-tumoral liver parenchyma or total LPT than for the tumor compartment. Therefore, ^99*m*^Tc-MAA-based activity planning using safety threshold could be used for SIRT planning before treatment to increase the tumor dose while avoiding overdosing of the normal liver parenchyma.

## Supplementary information

Additional file More examples of pre- and post-treatment VOI segmentation and dosimetry.

## Data Availability

The datasets of the images supporting the conclusions of this article may be made available upon request from the corresponding author.
