# Supplementary material for: Quantitative comparison of pre-treatment predictive and post-treatment measured dosimetry for selective internal radiation therapy using cone-beam CT for tumor and liver perfusion territory definition
Source: EJNMMI Res. 2020 Aug 14;10:94. doi: 10.1186/s13550-020-00675-5 (PMC7427681; doi:10.1186/s13550-020-00675-5)
Supplement: Supplementary file 1 — More examples of pre- and post-treatment VOI segmentation and dosimetry. [file 13550_2020_675_MOESM1_ESM.pdf]

Supplementary material

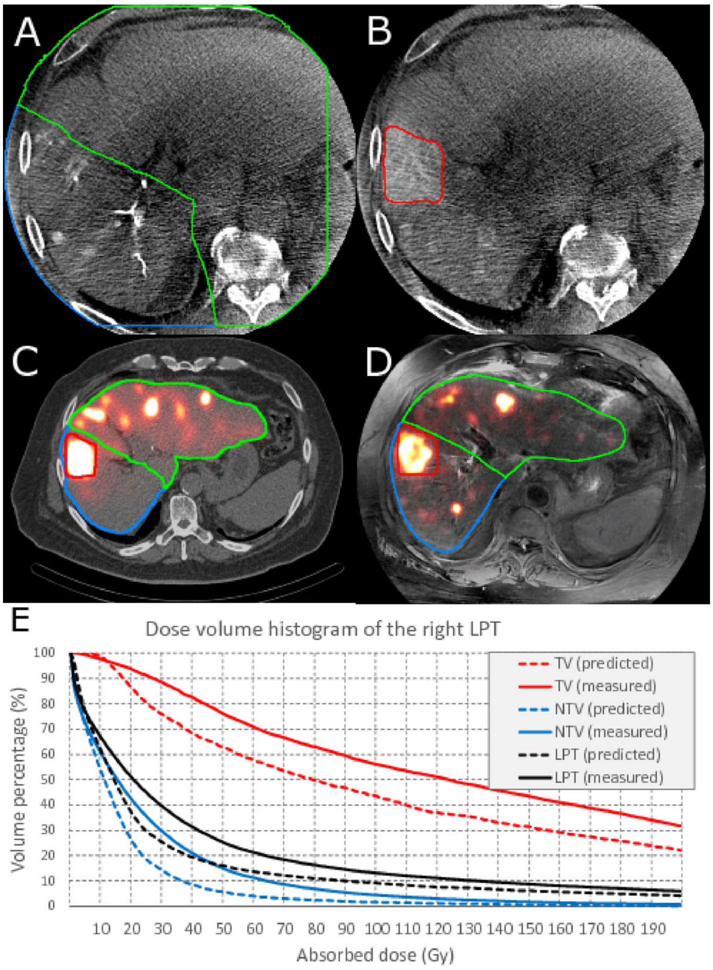

Figure 1: Single transaxial slice of a pre- and post-treatment study and dosimetry results (patient ID 26): [**A and B**] early and late arterial phase CBCTs focusing on the right LPT, the contrast enhancement is used to segment the right LPT (blue line) in the early phase image and to segment the tumor (red contour) on the late phase. [**C and D**] a fusion-view of ^{99m}Tc-MAA SPECT/CT and ^{90}Y PET/MR images. The contours are delineated on CBCT, transferred to CT (non-rigid registration), and masked by the total liver (blue, green, and red contours correspond to the right, left LPTs, and tumor). [**E**] dose-volume histogram of the tumor, non-tumoral liver parenchyma, and total LPT from predictive dosimetry (tumor, non-tumoral tissue, and total LPT mean dose of 113, 17 and 34 Gy) and measured doses (tumor, non-tumoral tissue, and total LPT mean dose of 149, 27 and 47 Gy).

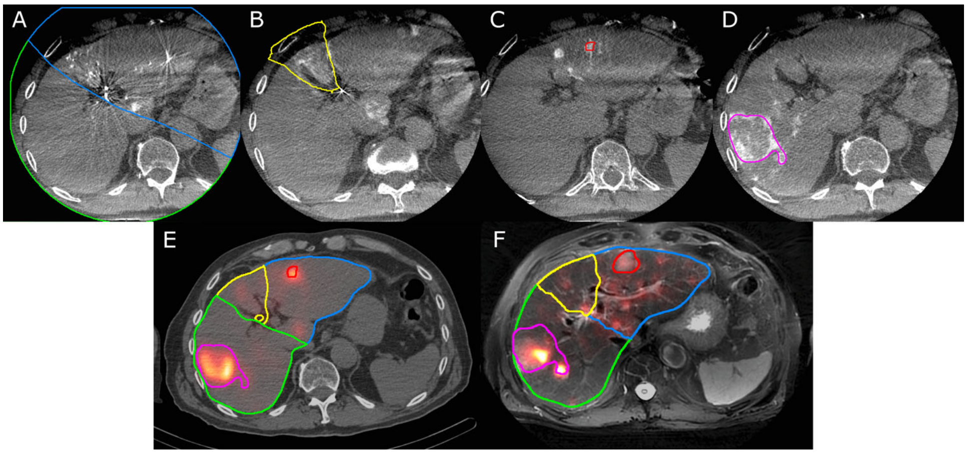

Figure 2: An example of the role of CBCT in VOI segmentation for super-selective LPTs: single transaxial slice of a pre- and post-treatment studies (patient ID 46):[**A and B**] an early arterial phase of CBCT focusing on the right and middle LPT and a late arterial phase of CBCT focusing on the middle LPT, the contrast enhancement is used to segment three compartments. [**C and D**] the late arterial phase of CBCT focusing on the right and left LPT, the contrast enhancement is used to delineate tumors. [**E and F**] a fusion-view of ^{99m}Tc-MAA SPECT/CT and ^{90}Y PET/MR images. The contours represent registered VOIs delineated on CBCTs masked by the total liver.

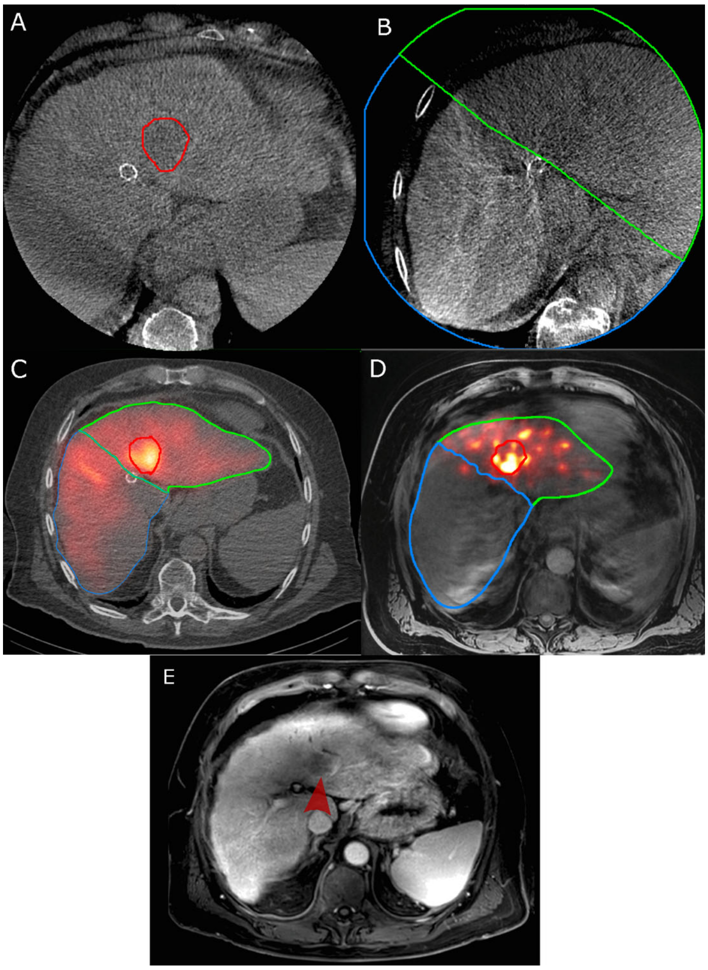

Figure 3: An example of the role of CBCT in VOI segmentation. In this case, CBCT-based LPT segmentation (left lobe) matched with ^{90}Y activity deposition. This confirms that our workflow performed well for this case. Single transaxial slice of a pre- and post-treatment studies (patient ID 16): [**A**] a late arterial phase of CBCT focusing on the left liver LPT, the contrast enhancement is used to segment the tumor (red contour). [**B**] a late arterial phase of CBCT focusing on the right liver LPT, the contrast enhancement is used to segment the right and left LPT (blue and green area). [**C and D**] a fusion-view of ^{99m}Tc-MAA SPECT/CT and ^{90}Y PET/MR images. The contours represent registered VOIs delineated on CBCTs masked by the total liver. During the simulation, ^{99m}Tc-MAA were injected in both liver lobes, but during the therapy session, microspheres were administered within the left lobe only. One can see the correspondence of CBCT based LPT segmentation and the volume infused with microspheres on the post-treatment ^{90}Y-PET images. [**E**] A baseline MR image, the red arrow point to the tumor which has been delineated on CBCT (figures A)
